# The Impact of Perfluoroalkyl Groups on Phosphane Basicity

**DOI:** 10.3390/molecules30102220

**Published:** 2025-05-20

**Authors:** Marta-Lisette Pikma, Aleksander Trummal, Ivo Leito, Agnes Kütt

**Affiliations:** 1 Institute of Chemistry, University of Tartu, Ravila 14a, 50411 Tartu, Estonia; marta.pikma@ut.ee (M.-L.P.); ivo.leito@ut.ee (I.L.); 2 National Institute of Chemical Physics and Biophysics, 23 Akadeemia Tee, 12618 Tallinn, Estonia; aleksander.trummal@kbfi.ee

**Keywords:** phosphanes, perfluoroalkyl group, basicity, COSMO-RS, DFT calculations, SMD model

## Abstract

This study employed computational methods to investigate the basicity of a series of polyfluorinated phosphanes. Results revealed an exceptionally low basicity, with the computed p*K*_aH_ values in acetonitrile approaching −30, a value significantly lower than anticipated. The good agreement between the SMD and COSMO-RS methods provided confidence in the reliability of these values. This unexpected behavior challenges conventional perceptions of phosphane basicity and deepens our understanding of the electronic effects of fluorination. The findings hold important implications for catalysis, ligand design, and main-group chemistry, where a precise comprehension of phosphane electronic properties is crucial. p*K*_aH_(MeCN) values, gas-phase basicities, and steric parameters are reported for 14 phosphanes.

## 1. Introduction

Tertiary phosphanes (PR_3_) are among the most widely utilized ligands in coordination chemistry [1,2,3,4]. Their popularity stems from the ability to fine-tune their steric and electronic properties through substituent modifications. When used as ligands, all PR_3_ compounds function as both σ-donors and π-acceptors [5]. However, in most cases, σ-donation is the predominant effect, particularly when the substituents exhibit a positive field-inductive effect (I+). Replacing these groups with those possessing a negative field-inductive effect (I−) enhances the contribution of π-bonding in the metal complex, thereby modifying the electronic properties of the phosphane [5]. Depending on the nature of the substituent, phosphanes can be electron rich (e.g., P(2,4,6-(MeO)_3_-C_6_H_2_)_3_), electron neutral (e.g., PPh_3_), or electron poor (e.g., P(C_2_F_5_)_3_).

Electron-rich phosphanes have garnered significant attention due to their highly nucleophilic nature, but recently, interest in electron-deficient phosphanes has also increased. Historically, carbon monoxide was the primary electron-withdrawing ligand, possessing unparalleled π-acceptor strength in tunable alternatives. However, the development of accessible synthesis methods for preparing phosphanes with perfluoroalkyl (Rf) groups has introduced new electron-withdrawing ligands [5,6,7,8]. Electron-withdrawing phosphane ligands play a crucial role in adjusting both the redox potential and the Lewis acidity of the corresponding transition metal complexes. These ligands can be used to promote metal-mediated reductive elimination and accelerate coupling reactions, in addition to serving as perfluorinating reagents [9].

Perfluorinated alkylphosphanes, including P(CF_3_)_3_ (**11**), P(C_2_F_5_)_3_ (**12**), P(C_3_F_7_)_3_ (**13**), and P(C_4_F_9_)_3_ (**14**), exhibit unique electronic and steric properties [5,10]. The presence of fluorinated alkyl groups renders these phosphanes highly electron-deficient, thereby reducing the basicity and nucleophilicity of the phosphorus atom. Despite their weak basicity, some perfluoroalkyl-containing phosphanes (e.g., (CH_3_)_2_PCF_3_) can still interact with strong Lewis acids like BF_3_. Their binding affinity is lower than that of conventional phosphanes (e.g., PMe_3_) [11], meaning they form weaker bonds with Lewis acids and transition metals, making them useful in catalysis where controlled electron donation is needed [12].

Additionally, the steric bulk introduced by perfluoroalkyl groups influences their coordination behavior in metal complexes. These properties make perfluoroalkylphosphanes valuable ligands and reagents in modern synthetic chemistry [13]. Due to their distinctive steric and electronic characteristics, perfluoroalkylphosphanes are essential in homogeneous catalysis [14,15], including hydroformylation [16], hydrogenation [17,18], and cross-coupling reactions [19,20,21], as well as in organometallic chemistry for stabilizing metal complexes [22,23]. They also play a role in the synthesis of fluorinated materials [24,25] and have applications in electronics and pharmaceuticals [9].

In a recent work, we suggested using the p*K*_aH_ value (in acetonitrile) and CPC angle, or exact cone angle (*θ*_H_), of a phosphane to assess its electronic and steric properties, respectively [26]. All these quantities are relatively easy to obtain from protonated phosphanes, using either experimental or computational methods, without involving metal–ligand complexes as a prerequisite. The calculations in that work were carried out employing the conductor-like screening model for realistic solvation (COSMO-RS), which is a hybrid of the dielectric continuum solvation model and statistical thermodynamics. Despite the advantages of COSMO-RS [27], our experience suggests that, while it is effective for obtaining relative results, it is not the most reliable method for determining the absolute p*K*_aH_ values for phosphanes without correction via correlation with experimental results. Additionally, each new version of the COSMOtherm software (25.0.0) introduces updated parameterizations, which can significantly impact the results. As demonstrated previously [26], the accuracy of COSMO-RS predictions is strongly dependent on the chosen parameterization. In the case of phosphanes, using older parameterizations (2017 and earlier) leads to significantly better agreements with the experiment compared to more recent parametrizations.

Other dielectric continuum methods (PCM [28], CPCM [29,30], IPCM [31], IEF-PCM [32,33,34,35], SMD [36], SM8 [37], SVPE [38,39,40]) have also been widely employed for calculating the basicity and acidity of compounds in water [41], as well as different organic solvents [42,43].

It has been suggested that Gibbs free energy of solvation is influenced not only by bulk polarization effects but also by first-layer solvation effects arising from short-range solute–solvent interactions [44]. These effects can be accounted for by explicitly incorporating solvent molecules into the solute structure before applying a dielectric continuum model in a discrete–continuum approximation. Apart from aqueous solutions being usually characterized by strong specific solvation effects [36,44], the inclusion of explicit solvent molecules has also been proven to be beneficial in highly polar organic solvents, such as DMSO, as has been demonstrated by Wang et al. [43]—at least when paired with the polarizable continuum model (PCM). In contrast, for solvents with lower dielectric constants, such as acetonitrile (MeCN) and tetrahydrofuran (THF), the implicit PCM solvation generally provides more accurate results. Additionally, in the case of protonated phosphanes, the charge of the solute ions is significantly delocalized, resulting in weaker solute–solvent interactions. Based on these considerations, explicit solvent molecules were not included in the calculations for p*K*_aH_(MeCN) values in this work.

The purpose of this study was to predict p*K*_aH_ values of fluorinated phosphanes, assess the impact of perfluoroalkyl groups on phosphane basicity using the SMD solvent model, and compare the results to the corresponding COSMO-RS values.

## 2. Results and Discussion

Altogether, 14 fluorinated phosphanes were investigated. The p*K*_aH_(MeCN) values calculated using the SMD (single-point (SP) calculations, see below) and COSMO-RS models, along with the available experimental p*K*_aH_(MeCN) values, calculated gas-phase basicity (GB) values, Tolman electronic parameters, CPC angles, exact cone angles, and Tolman cone angles, are presented in Table 1. See also Supplementary Materials. 

As is seen from Table 1 and ref. [26], electron-withdrawing groups (e.g., F, CF_3_) reduce the basicity of aromatic phosphanes, while electron-donating groups (e.g., MeO) enhance it. This is illustrated by comparing compounds **2**, **4**, and **6**–**7** with the methoxy-substituted aromatic phosphanes reported in ref [26]. The p*K*_aH_ values of the former range from 2.5 to 6.5 in MeCN, whereas the methoxy-substituted derivatives P(4-MeO-C_6_H_4_)_3_, P[2,4,6-(MeO)_3_-C_6_H_2_]Ph_2_, P[2,6-(MeO)_2_-C_6_H_3_]_3_, P[2,4,6-(MeO)_3_-C_6_H_2_]_2_Ph, and P[2,4,6-(MeO)_3_-C_6_H_2_]_3_ exhibited significantly higher p*K*_aH_ values, ranging from 10 to 20 [26]. Certain phosphanes with a P(N=CX_2_)_3_ structural motif can even exhibit p*K*_aH_ values approaching 40 in MeCN [50].

The decrease in basicity caused by electron-withdrawing substituents is primarily attributed to the destabilization of the protonated form, as these groups increase the positive charge density on the phosphorus atom, thereby making the phosphane less prone to protonation. Moreover, although conjugation between the phosphorus lone pair and the aromatic ring is limited due to unfavorable orbital alignment, a minor resonance effect persists. In aromatic phosphanes, electron-withdrawing groups can therefore decrease the availability of the phosphorus lone pair, further contributing to reduced basicity. The effect is reversed for electron-donating groups, which increase the basicity of phosphanes by stabilizing the protonated form and enhancing the availability of the phosphorus lone pair.

The difference between experimental and SMD-calculated p*K*_aH_ values of arylphosphanes (compounds **2**, **4**, **6**–**7**) is relatively small, with the largest deviation being only 0.2 units. This highlights one advantage of the SMD method over COSMO-RS, as SMD does not require a correlation equation—using one reference base alone is sufficient. In contrast, without applying any empirical correlation, the COSMO-RS values computed using parameterizations from 2018 onward differ from the experimental results by 4–6 units, whereas the 2017 parameterization reduces this discrepancy to approximately 1 unit. When implementing the SMD model without a reference base, and instead the Gibbs free energy of proton solvation is used, the deviation from experimental results is smaller (0.4–0.6 units) compared to COSMO-RS. These deviations were observed using the Gibbs free energy of proton solvation of −252.39 kcal/mol reported by Himmel [51], which was selected because it resulted in calculated p*K*_aH_ values that exhibited the optimal agreement with experimental results.

**Figure 1:**
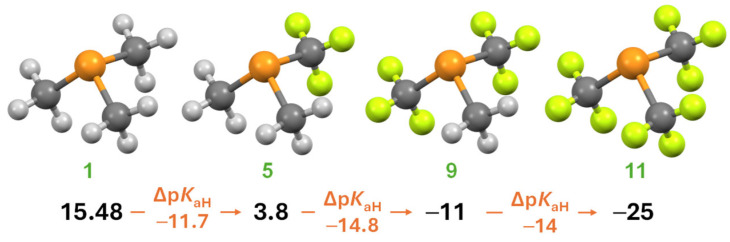
The impact of replacing Me groups with CF_3_ groups on p*K*_aH_(MeCN) values. p*K*_aH_ values in black are from Table 1, and corresponding compound numbers are marked in green below the structures. The differences in p*K*_aH_ values (Δp*K*_aH_) between compounds are in orange on the arrows.

As shown in references [26,47], alkyl-substituted phosphanes generally exhibit higher basicity compared to aromatic phosphanes, with the exception of aromatic phosphanes that contain predominantly methoxy-substituted aromatic rings. This is attributed to the negative field-inductive effect of the phenyl ring, which destabilizes the protonated form of the phosphane, as previously discussed. Similarly, fluorination of the alkyl groups has a decreasing effect on phosphane basicity due to the strong electron-withdrawing nature of fluorine. Replacing a methyl group with a trifluoromethyl group in PMe_3_ reduces its basicity by approximately 11–15 p*K*_aH_ units per methyl group, as illustrated in Figure 1. This substantial change highlights the distinctive properties of fluorine that result from its exceptionally high electronegativity.

The difference between the experimental and calculated (SMD model) p*K*_aH_ values is notably larger for PMe_3_ compared to the other phosphanes examined in this work. This may arise from the use of fluorinated arylphosphane as the reference base and the low p*K*_aH_ value of the reference base compared to PMe_3_.

This discrepancy is probably also present in the CF_3_-substituted derivatives, as well as in other perfluoroalkylphosphanes **12**–**14**. Nevertheless, this leads to estimates of p*K*_aH_ values in MeCN for tris-perfluoroalkyl phosphanes that are below −20, which are the lowest predicted for any phosphane. In addition, it is important to note that these values involve significant extrapolation from the experimentally accessible basicity domain, so that their uncertainties most likely amount to several p*K*_aH_ units. However, even with such uncertainties, these values are informative. The close alignment of p*K*_aH_ values between the SMD and COSMO-RS methods, as well as the low GB values, suggests that these p*K*_aH_ values are reasonably accurate.

Overall, p*K*_aH_ values below −20 indicate a negligible basicity, suggesting that P(Rf)_3_ compounds do not behave as Brønsted bases under almost any realistic experimental conditions. However, due to the strong π-backbonding effect induced by electronegative substituents, they exhibit significantly greater π-acidity compared to conventional PR_3_ compounds, as the energy of their σ* anti-bonding orbitals is lower. This effect significantly stabilizes the resulting transition metal complexes, while the proton is not a subject of the π-backbonding.

In our previous work [26], we suggested Equation (1) to express the correlation between p*K*_aH_(MeCN) and TEP values:

TEP = −1.35(0.05)·p*K*_aH_ + 2080(1) (1)

*n* = 20; *R*^2^ = 0.977; *S* = 1.29

Applying Equation (1) to phosphanes **10**–**12** results in p*K*_aH_(MeCN) values that are higher than those calculated with the SMD method (Table 1). p*K*_aH_(MeCN) values from Equation (1) are −23, −18 and −17, respectively. While the difference is smaller for PF_3_ (under 1 unit), it is significantly larger for P(CF_3_)_3_ and P(C_2_F_5_)_3_ – at 7 and 11 units, respectively. This discrepancy may arise from the fact that the TEP values for the latter two compounds are calculated rather than experimentally determined, or it may be attributed to differences in π-backbonding. The steric bulk of P(CF_3_)_3_ and P(C_2_F_5_)_3_ likely restricts how closely they can interact with the metal center, compared to PF_3_. Additionally, the correlation (1) is not ideal, as certain outliers (PMe_3_, P(2-MeO-C_6_H_4_)_3_ and P(dma)_3_) were omitted based on Grubbs’ test.

**Scheme 1:**
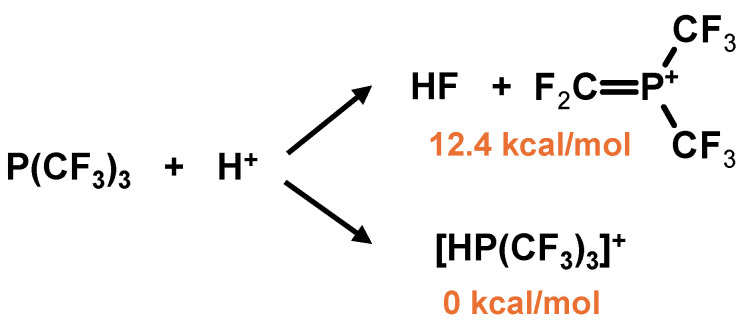
Different protonation pathways of P(CF_3_)_3_ and the corresponding relative gas-phase energies of the resulting species (MP2/6311++G(d,p)//M06-2X/6-31+G(d)).

The extremely low basicity of the phosphorus basicity center of compounds **11**–**14** leads us to question whether protonation of some other basicity center could compete with the P center, specifically through protonation at the fluorine atom. Computational studies from this work indicate that fluorine-protonated species undergo in silico decomposition into HF and a corresponding cation. The resulting cation, together with dissociated HF, exhibits a gas-phase relative energy increase of approximately 12 to 18 kcal/mol (8 to 13 orders of magnitude of the equilibrium constant) compared to when protonation occurs at the phosphorus center (Scheme 1), indicating that fluorine protonation is still unlikely.

## 3. Computational Methods

Gas-phase basicity (GB) is defined as the Gibbs free energy (*G*) of the following proton abstraction equilibrium (2) in the gas phase:

(2) $${\mathrm{BH}}^{+}\;\begin{array}{c} \mathrm{GB}\\ ⇄ \end{array}\;B+H^{+}$$

where BH^+^ is the conjugated acid of base B. The method used for calculating GB is described in ref [52].

**Scheme 2:**
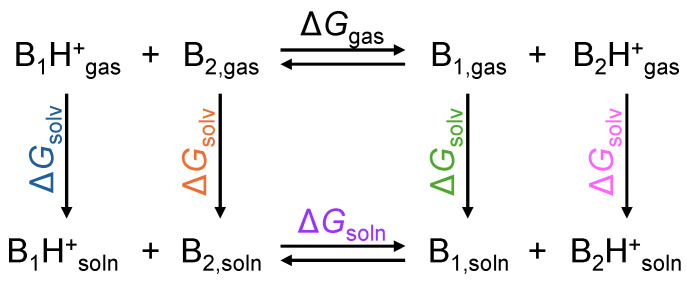
Thermodynamic cycle of proton exchange between the bases B_1_ and B_2_.

The relative p*K*_aH_ calculations in MeCN were carried out based on the proton exchange thermodynamic cycle shown in Scheme 2, following a methodology similar to that employed in previous studies [42,43]. We prefer this approach over the direct thermodynamic cycle, as it does not rely on the absolute value of the Gibbs free energy of solvation of proton in MeCN, which is known to have significant uncertainty [53]. Instead, one base with a well-established experimental p*K*_aH_ is chosen as the reference (or anchor) base B_2_, and the p*K*_aH_ of the studied base B_1_ is determined according to the following expression (3) and the corresponding expansion for Δ*G*_soln_ (4):

(3) $$pK_{\mathrm{aH}}(B_{1})=pK_{\mathrm{aH}}(B_{2})+\frac{{∆G}_{\mathrm{soln}}}{RT\ln\left(10\right)}$$

(4) $${∆G}_{\mathrm{soln}}=\mathrm{GB}(B_{1})+\;{\Delta G}_{\mathrm{solv}}\left(B_{1}\right)-{\Delta G}_{\mathrm{solv}}(B_{1}H^{+})\;-\mathrm{GB}(B_{2})\;-{\Delta G}_{\mathrm{solv}}\left(B_{2}\right)+{\Delta G}_{\mathrm{solv}}(B_{2}H^{+})$$

Here, p*K*_aH_ stands for the negative logarithm of the specified base’s conjugated acid’s dissociation constant *K*_a_ in solution; GB refers to the gas-phase basicity of the respective base; Δ*G*_soln_ is the Gibbs free energy of the proton exchange between bases B_1_ and B_2_ in solution; and Δ*G*_solv_ denotes the Gibbs free energy of solvation of the specified species. P(2,6-F_2_-C_6_H_3_)Ph_2_ (experimental p*K*_aH_ = 5.17) was used as the reference base B_2_ in this work. This base was chosen as a reference due to the presence of fluorine atoms in its structure and because it is not positioned at the lowest end of the p*K*_aH_ scale, where measurements tend to be less precise owing to the substantial amount of acid required for protonation.

The conformational search in the gas phase (at BP86/def-TZVP level of theory) for each compound was performed with COSMOconf 24.1.0, and the conformer with the lowest COSMO energy was then selected for all further calculations. The respective structures were subsequently reoptimized in the gas phase and in solution with Gaussian 16 using the same density functional and basis set combination (M06-2X/6-31+G(d)). Reaching true minima on potential energy surface was confirmed by inspecting the vibrational normal modes’ eigenvalues from the frequency calculations. The SMD solvation model was used, as it is the recommended choice for calculating Gibbs free energies of solvation (Δ*G*_solv_) with Gaussian software package [36]. The Δ*G*_solv_ values were obtained as the differences in SCF energies of the structure in the gas phase and in solution, where the latter quantity includes both electrostatic and non-electrostatic (CDS) terms according to the SMD definition. For obtaining GB values, an additional single-point calculation was done at the MP2/6-311++G(d,p) level of theory, while the thermal correction was extracted from the M06-2X/6-31+G(d) calculation.

For some protonated phosphanes (i.e., [HP(CF_3_)_3_]^+^ and [HP(C_6_F_5_)_3_]^+^) the SMD optimizations using a default solute cavity based on intrinsic atomic Coulomb radii and their corresponding scaling factor consistently resulted in remarkable energy oscillations. The likely root cause is the complicated electrostatic pattern that arises due to shielding of the partial charge on phosphorus by the presence of multiple fluorine atoms or fluorinated phenyl rings nearby, especially in the case of the cations, where significant steric shielding occurs directly around the phosphorus atom. Consequently, for these structures, the Δ*G*_solv_ was obtained by subjecting the gas-phase optimized structures to single-point (SP) SMD calculations at the M06-2X/6-31+G(d) level of theory. This approach was subsequently extended to all studied compounds to compare the SP results with those obtained from solvent-optimized structures (see Supplementary Materials). Although the Δ*G*_solv_ varied significantly for some structures, the mean absolute error (MAE) of the p*K*_aH_ values was 0.4 units. The largest deviations were observed for P(CF_3_)_2_Me, PF_3_, P(C_3_F_7_)_3_, and P(C_4_F_9_)_3_, with the highest discrepancy reaching 1.2 p*K*_aH_ units for the latter. Interestingly, the SP results generally exhibited better alignment with experimental p*K*_aH_ values, leading to their selection for use in this study.

The calculations for the COSMO-RS p*K*_aH_ values, CPC angles, and exact cone angles were carried out following the methods described in refs [26,52]. For COSMO-RS calculations, the structures were first optimized at the BP86/def-TZVP level (reaching the true minima was ensured by subsequent frequency calculations), followed by a single-point calculation using the def2-TZVPD basis set of the same functional, COSMO model, and Fine cavity parameter. The parametrization BP_TZVPD_FINE_C30_1701 was used to calculate the p*K*_aH_(Calc) values in MeCN, which were then corrected using the correlation Equation (5) [26] to derive the predicted p*K*_aH_(MeCN) values presented in this work, as follows:

(5) $${pK}_{\mathrm{aH}}\left(\mathrm{Calc}\right)=1.02\left(0.02\right)\;\cdot \;{pK}_{\mathrm{aH}}\left(\mathrm{Exp}\right)-0.98(0.3)$$

*n* = 25; *R*^2^ = 0.987; *S* = 0.685

The calculations were carried out using the following software: Gaussian 16 [54], Turbomole 7.7 [55], Avogadro 1.1.1, Avogadro 1.95.0, Tmolex 24.1.0 [56], COSMOconf 24.1.0 [57], COSMOtherm 25.0.0 [58], R 4.4.3, and RStudio 2024.12.1 [59].

## 4. Conclusions

The SMD and COSMO-RS solvation models were employed to calculate the p*K*_aH_(MeCN) values of fluorinated phosphanes, including perfluoroalkylphosphanes. In contrast to the COSMO-RS approach, the SMD model, when applied to proton exchange equilibria involving a reference base, provided reliable p*K*_aH_(MeCN) predictions without requiring any empirical correlations. Notably, the SMD method exhibited limited accuracy in the case of the only non-fluorinated compound (PMe_3_), likely due to the selection of an arylphosphane P(2,6-F_2_-C_6_H_3_)Ph_2_ as the reference base. The exceptionally low computed p*K*_aH_ values of the perfluoroalkylphosphanes indicate that these compounds have a negligible basic character, instead demonstrating significant π-accepting properties of the perfluoroalkyl groups.

As demonstrated, the basicity of phosphanes can be effectively fine-tuned by varying their substituents, enabling coverage of a wide p*K*_aH_ range (from approximately −30 to over 30 p*K*_aH_ units in acetonitrile). Incorporation of electron-accepting groups leads to a decrease in the p*K*_aH_ value, whereas the introduction of electron-donating groups has the opposite effect, resulting in increased basicity.

## Figures and Tables

**Table 1:** p*K*_aH_(MeCN) (unitless value) and GB values in kilocalories per mole (kcal·mol^−1^); CPC (*θ*_CPC_) and exact cone angles (*θ*_H_) in degrees (°); Tolman electronic parameters (TEP) expressed as the wavenumber (cm^−1^) of the A_1_ C-O vibrational mode of the [LNi(CO)_3_] complex, where L is the phosphane [45,46]; and Tolman cone angles (*θ*_Tolman_) in degrees (°) [10,45] of phosphanes. p*K*_aH_(MeCN) values calculated with the SMD method are from single-point calculations at the M06-2X/6-31+G(d) level of theory using structures optimized in the gas phase at the same theoretical level. For GB values, the structure was first optimized using the M06-2X functional with a 6-31+G(d) basis set, and an additional single-point calculation was done at the MP2/6-311++G(d,p) level of theory; thermal correction was obtained from M06-2X/6-31+G(d) calculation.

No	Compound	p*K*_aH_(MeCN)			GB [kcal·mol^−1^]	TEP [cm^−1^]	*θ*_CPC_ [°] *^b^*	*θ*_H_ [°] *^b^*	*θ*_Tolman_ [°]
		Experimental	SMD	COSMO-RS *^a^*					
**1**	PMe_3_	15.48 *^c^*	18.5	15.6 *^f^*	220.3	2064.1	110.4	163	118
**2**	P(2-F-C_6_H_4_)Ph_2_	6.11 *^d^*	6.3	5.9 *^f^*	221.1		110.8	220	
**3**	P(2,6-F_2_-C_6_H_3_)Ph_2_	5.17 *^d^*	*5.17 ^e^*	5.5 *^f^*	220.7		111.4	221	
**4**	P(2-F-C_6_H_4_)_2_Ph	4.56 *^d^*	4.6	4.5 *^f^*	219.4		110.5	226	
**5**	P(CF_3_)Me_2_		3.8	1.7	196.5		109.8	169	124
**6**	P(C_6_F_5_)Ph_2_	2.54 *^d^*	2.7	2.7 *^f^*	212.7	2074.8	111.6	222	158
**7**	P(2,6-F_2_-C_6_H_3_)_2_Ph	2.5 *^d^*	2.4	2.5 *^f^*	217.0		111.7	232	
**8**	P(C_6_F_5_)_3_		−8	−7 *^f^*	190.4	2090.9	112.6 *^g^*	256 *^g^*	184
**9**	P(CF_3_)_2_Me		−11	−13	174.4		109.0	174	
**10**	PF_3_		−24	−23	146.2	2110.8	108.0	149	104
**11**	P(CF_3_)_3_		−25	−27	156.4	2104.4	109.0 *^g^*	185 *^g^*	137
**12**	P(C_2_F_5_)_3_		−29	−27	158.8	2102.9	109.0	217	171
**13**	P(C_3_F_7_)_3_		−28	−27	162.1		110.2	227	
**14**	P(C_4_F_9_)_3_		−32	−28	159.8		110.3	215	

*^a^* Correlated values using Equation (5). This work, unless indicated otherwise. *^b^* Obtained from solvent-phase optimized cations (SMD/M06-2X/6-31+G(d)). *^c^* Experimental value initially published in ref [47], which was later slightly revised in ref [48]. *^d^* Experimental value initially published in ref [49], which was later slightly revised in ref [48]. *^e^* Experimental value [48] used as a reference. *^f^* Value from ref [26]. *^g^* Value from gas-phase geometry.

## Data Availability

The data that support the findings of this study are available in the ESI of this article and in DataDOI repository at https://doi.org/10.23673/re-508 [61].

## Supplementary Materials

The following supporting information can be downloaded at: https://www.mdpi.com/article/10.3390/molecules30102220/s1, Table S1: Calculated energies for phosphanes **1**–**14**; Cartesian coordinates for the gas-and solvent-phase structures.
