# Supplementary material for: The Impact of Perfluoroalkyl Groups on Phosphane Basicity
Source: Molecules. 2025 May 20;30(10):2220. doi: 10.3390/molecules30102220 (PMC12114529; doi:10.3390/molecules30102220)
Supplement: Supplementary file 1 [file molecules-30-02220-s001.zip › molecules-3622960-supplementary.pdf]

Contents

Computational methods S2
Calculation of pK_{aH} values with SMD model S2
Cartesian coordinates for gas-phase structures S3
Cartesian coordinates for solvent-phase structures (SMD model) S10

Computational methods

Calculation of pK_{aH} values with SMD model

The geometries (.xyz files) for all the calculated species are available at DataDOI (https://doi.org/10.23673/re-508) [61].

Table S1. Calculated energies for phosphanes **1-14**.

No	Compound	Form	G(gas)[a] [AU]	GB[a] [kcal/mol]	SCF(gas)[b] [AU]	SCF(MeCN)[c] [AU]	ΔG_solv[c] [kcal/mol]	SCF(MeCN)[d] [AU]	ΔG_solv[d] [kcal/mol]
**1**	PMe_3	Neutral	-460.1324	220.3	-460.9945	-460.9880	4.1	-460.9985	-2.5
		Cation	-460.4935		-461.3588	-461.4424	-52.5	-461.4551	-60.4
**2**	$P(2\text{-}F\text{-}C_6H_4)Ph_2$	Neutral	-1132.7256	221.1	-1135.2120	-1135.2159	-2.5	-1135.2342	-13.9
		Cation	-1133.0879		-1135.5820	-1135.6483	-41.6	-1135.6684	-54.2
**3**	$P(2,6\text{-}F_2\text{-}C_6H_3)Ph_2$	Neutral	-1231.8065	220.7	-1234.4185	-1234.4244	-3.7	-1234.4413	-14.3
		Cation	-1232.1682		-1234.7883	-1234.8546	-41.6	-1234.8734	-53.4
**4**	$P(2\text{-}F\text{-}C_6H_4)_2Ph$	Neutral	-1231.8126	219.4	-1234.4238	-1234.4291	-3.3	-1234.4461	-14.0
		Cation	-1232.1722		-1234.7913	-1234.8580	-41.9	-1234.8768	-53.7
**5**	$P(CF_3)Me_2$	Neutral	-757.4093	196.5	-758.6442	-758.6421	1.3	-758.6493	-3.2
		Cation	-757.7324		-758.9692	-759.0632	-59.0	-759.0734	-65.4
**6**	$P(C_6F_5)Ph_2$	Neutral	-1529.0352	212.7	-1532.0183	-1532.0242	-3.7	-1532.0372	-11.8
		Cation	-1529.3841		-1532.3741	-1532.4480	-46.4	-1532.4632	-55.9
**7**	$P(2,6\text{-}F_2\text{-}C_6H_3)_2Ph$	Neutral	-1429.9768	217.0	-1432.8386	-1432.8464	-4.9	-1432.8609	-14.0
		Cation	-1430.3326		-1433.2020	-1433.2702	-42.9	-1433.2865	-53.1
**8**	$P(C_6F_5)_3$	Neutral	-2519.8295	190.4	-2524.0531	-2524.0630	-6.2	–	–
		Cation	-2520.1430		-2524.3691	-2524.4596	-56.7	–	–
**9**	$P(CF_3)_2Me$	Neutral	-1054.6758	174.4	-1056.2821	-1056.2827	-0.4	-1056.2867	-2.8
		Cation	-1054.9637		-1056.5700	-1056.6698	-62.6	-1056.6769	-67.1
**10**	PF_3	Neutral	-639.9826	146.2	-640.8458	-640.8485	-1.7	-640.8488	-1.9
		Cation	-640.2256		-641.0881	-641.2072	-74.7	-641.2078	-75.1
**11**	$P(CF_3)_3$	Neutral	-1351.9272	156.4	-1353.9088	-1353.9094	-0.4	–	–
		Cation	-1352.1865		-1354.1623	-1354.2607	-61.8	–	–
**12**	$P(C_2F_5)_3$	Neutral	-2063.9631	158.8	-2067.0663	-2067.0645	1.1	-2067.0647	1.0
		Cation	-2064.2261		-2067.3276	-2067.4117	-52.8	-2067.4145	-54.6
**13**	$P(C_3F_7)_3$	Neutral	-2775.9984	162.1	-2780.2220	-2780.2191	1.8	-2780.2193	1.7
		Cation	-2776.2668		-2780.4867	-2780.5648	-49.1	-2780.5688	-51.5
**14**	$P(C_4F_9)_3$	Neutral	-3488.0313	159.8	-3493.3769	-3493.3726	2.7	-3493.3729	2.5
		Cation	-3488.2960		-3493.6435	-3493.7153	-45.0	-3493.7200	-48.0

[a] MP2/6311++G(d,p)//M062X/6-31+G(d). [b] M062X/6-31+G(d). [c] From single point calculations; SMD/M062X/6-31+G(d). [d] From solvent optimized structures; SMD/M062X/6-31+G(d).

<u>Cartesian coordinates for gas-phase structures</u>

P_26_F2_C6H3_2Ph_gas
34

C	-0.26768	4.27150	-0.95030
C	-0.94190	3.19218	-1.52092
C	-0.88867	1.93470	-0.92467
C	-0.16501	1.74658	0.25634
C	0.49687	2.83666	0.83178
C	0.45447	4.09185	0.22715
P	0.01440	0.14217	1.15883
C	-1.37653	-0.84869	0.45643
C	-2.67299	-0.35279	0.62386
C	-3.82461	-1.01323	0.23063
C	-3.69533	-2.27265	-0.34926
C	-2.43626	-2.84149	-0.51140
C	-1.32023	-2.12687	-0.10064
F	-0.12382	-2.72707	-0.24371
F	-2.80618	0.85067	1.21441
C	1.50318	-0.49962	0.27226
C	1.60931	-0.71035	-1.10394
C	2.76859	-1.12135	-1.73734
C	3.90583	-1.33567	-0.95949
C	3.86869	-1.14275	0.41676
C	2.67375	-0.73157	0.99297
F	2.65931	-0.54360	2.32503
F	0.51298	-0.52320	-1.85976
H	-0.31059	5.24949	-1.41946
H	-1.50933	3.32785	-2.43652
H	-1.40906	1.09935	-1.38396
H	1.04465	2.70396	1.76211
H	0.97487	4.92836	0.68292
H	-4.78699	-0.54200	0.39129
H	-4.57894	-2.81646	-0.66471
H	-2.29723	-3.82545	-0.94360
H	2.76489	-1.26942	-2.81070
H	4.82740	-1.65697	-1.43285
H	4.73487	-1.30120	1.04805

P_26_F2_C6H3_2Ph_H_gas
35

C	-1.24862	4.42680	-0.03222
C	-2.05735	3.57490	-0.78778
C	-1.66900	2.25848	-1.00239
C	-0.46152	1.80710	-0.45408
C	0.35312	2.65245	0.30187
C	-0.04968	3.96961	0.50989
P	0.02250	0.10990	-0.75559
C	1.62686	-0.27914	-0.06720
C	2.76637	-0.37216	-0.87196
C	4.00957	-0.69691	-0.36061
C	4.11698	-0.93504	1.00909
C	3.01119	-0.85123	1.85728
C	1.79122	-0.52352	1.29937
F	0.70197	-0.42244	2.07221
F	2.62916	-0.13158	-2.18113
C	-1.22500	-1.05988	-0.22381
C	-1.38015	-2.26401	-0.92045
C	-2.32874	-3.20917	-0.58423
C	-3.15722	-2.94229	0.50635
C	-3.03911	-1.76408	1.24215
C	-2.07390	-0.84594	0.86856
F	-1.93898	0.28314	1.56571
F	-0.55500	-2.48616	-1.95470
H	-1.55729	5.45370	0.13289
H	-2.98902	3.93679	-1.20855
H	-2.30228	1.59402	-1.58544
H	1.28775	2.29592	0.72436
H	0.57397	4.63536	1.09617
H	4.86174	-0.75759	-1.02696
H	5.08486	-1.19042	1.42659
H	3.08385	-1.03186	2.92307
H	-2.40506	-4.12259	-1.16179
H	-3.90838	-3.67109	0.79090
H	-3.67213	-1.55010	2.09504
H	0.16004	-0.10307	-2.13026

P_26_F2_C6H3_Ph2_gas
34

C	-3.88685	-0.76354	1.14061
C	-2.71954	-0.53587	1.86685
C	-1.54180	-0.32079	1.16908
C	-1.44783	-0.31631	-0.22611
C	-2.65244	-0.55255	-0.89145
C	-3.86370	-0.77545	-0.24998
C	0.64912	1.59498	-0.40767
C	1.52274	1.67795	0.68199
C	1.90252	2.92032	1.18504
C	1.40788	4.09284	0.61440
C	0.53795	4.02020	-0.47148
C	0.17213	2.77770	-0.98609
C	1.25967	-1.21815	-0.51696
C	2.61635	-1.05519	-0.83312
C	3.55860	-1.99849	-0.43495
C	3.15705	-3.13429	0.26877
C	1.80985	-3.31498	0.56977
C	0.86685	-2.36304	0.18127
H	-4.82122	-0.93385	1.66463
H	-2.70482	-0.52295	2.95033
F	-2.65361	-0.56732	-2.23532
H	-4.75342	-0.95073	-0.84296
H	2.58152	2.97161	2.03073
H	1.70436	5.05876	1.01147
H	0.15489	4.92794	-0.92727
H	4.60578	-1.84957	-0.67951
H	3.88977	-3.87380	0.57587
H	1.48688	-4.19712	1.11404
F	-0.42351	-0.11980	1.88500
P	0.08561	0.02292	-1.20251
H	-0.48714	2.72450	-1.84964
H	1.90167	0.77060	1.14239
H	-0.17906	-2.52008	0.42916
H	2.93944	-0.17721	-1.38861

P_26_F2_C6H3_Ph2_H_gas

35

C	-4.05247	-0.80884	0.90462
C	-3.00152	-0.51786	1.77657
C	-1.75545	-0.28403	1.23016
C	-1.50762	-0.32488	-0.14495
C	-2.59428	-0.62172	-0.97229
C	-3.86269	-0.86374	-0.47520
C	0.67671	1.64290	-0.40546
C	2.03548	1.95861	-0.53989
C	2.45648	3.26189	-0.29808
C	1.53123	4.23687	0.07662
C	0.18101	3.91669	0.21073
C	-0.25475	2.61702	-0.03038
C	1.33469	-1.25339	-0.26916
C	2.00993	-1.11517	0.95042
C	2.91188	-2.10022	1.33828
C	3.13506	-3.20798	0.51890
C	2.46228	-3.33946	-0.69540
C	1.55845	-2.36060	-1.09728
H	-5.04028	-0.99763	1.31067
H	-3.13535	-0.47360	2.85079
F	-2.38611	-0.66899	-2.29503
H	-4.67037	-1.08878	-1.16146
H	3.50617	3.51496	-0.39912
H	1.86569	5.25134	0.26672
H	-0.53406	4.67705	0.50497
H	3.44157	-2.00265	2.27960
H	3.84172	-3.97134	0.82737
H	2.64448	-4.19826	-1.33202
F	-0.71527	0.00123	2.02765
P	0.13907	-0.02537	-0.78867
H	-1.30714	2.36979	0.07517
H	2.75826	1.19829	-0.82323
H	1.03916	-2.46126	-2.04675
H	1.83667	-0.25112	1.58524
H	-0.00657	-0.19307	-2.17073

P_2F_C6H4_2Ph_gas
34

C	-2.79915	-3.30703	0.72723
C	-2.28326	-3.44197	-0.55984
C	-1.46687	-2.44355	-1.09057
C	-1.14651	-1.30956	-0.33659
C	-1.67111	-1.18167	0.95496
C	-2.49472	-2.17391	1.48226
P	-0.01954	-0.07474	-1.11509
C	-0.46796	1.43819	-0.15562
C	-1.66046	2.07781	-0.49083
C	-2.10824	3.23682	0.12049
C	-1.32196	3.80142	1.12291
C	-0.12138	3.19681	1.49002
C	0.29665	2.02647	0.85744
C	1.57254	-0.52306	-0.29261
C	1.76420	-1.61981	0.55612
C	3.02855	-1.93216	1.05598
C	4.13027	-1.14857	0.71905
C	3.97189	-0.04971	-0.12379
C	2.70476	0.22716	-0.60848
F	2.55079	1.29768	-1.41722
H	-3.44132	-4.07887	1.14002
H	-2.52264	-4.31768	-1.15499
H	-1.07646	-2.54304	-2.10037
H	-1.43260	-0.30440	1.55098
H	-2.89815	-2.06311	2.48414
F	-2.42846	1.52835	-1.45523
H	-3.04987	3.67354	-0.19314
H	-1.65028	4.71152	1.61418
H	0.49173	3.63280	2.27169
H	1.23001	1.56068	1.15955
H	0.91282	-2.23701	0.82650
H	3.14942	-2.78835	1.71124
H	5.11396	-1.38842	1.10924
H	4.80198	0.58689	-0.40930

P_2F_C6H4_2Ph_H_gas
35

C	2.89300	-3.42221	0.53206
C	2.13184	-3.55542	-0.63039
C	1.26795	-2.53575	-1.01563
C	1.17654	-1.38320	-0.22542
C	1.93819	-1.24300	0.93960
C	2.79764	-2.27228	1.31484
P	0.01898	-0.09950	-0.69184
C	-1.63630	-0.52610	-0.15406
C	-2.71170	0.21251	-0.64770
C	-4.01878	-0.04541	-0.28044
C	-4.24904	-1.08465	0.62103
C	-3.19379	-1.84170	1.13437
C	-1.88567	-1.56804	0.74852
C	0.49365	1.49212	-0.02387
C	-0.20097	2.11474	1.02055
C	0.24627	3.33881	1.50610
C	1.37744	3.94192	0.95149
C	2.07965	3.33603	-0.08973
C	1.62134	2.11765	-0.55410
F	2.26522	1.49141	-1.55343
H	3.56900	-4.21843	0.82574
H	2.21578	-4.44893	-1.23924
H	0.67790	-2.63848	-1.92285
H	1.86889	-0.34144	1.54252
H	3.39610	-2.17258	2.21376
F	-2.43740	1.20515	-1.51128
H	-4.82227	0.55287	-0.69476
H	-5.26726	-1.30614	0.92278
H	-3.39180	-2.64795	1.83116
H	-1.06057	-2.15900	1.13592
H	-1.08417	1.64439	1.44361
H	-0.28881	3.82557	2.31324
H	1.71815	4.89901	1.33197
H	2.95775	3.78674	-0.53794
H	0.01260	-0.00835	-2.08938

P_2F_C6H4_Ph2_gas
34

C	1.11613	4.02541	0.86528
C	1.46052	2.82351	1.48161

C	1.12303	1.60785	0.88791
C	0.44267	1.58112	-0.33426
C	0.11329	2.79375	-0.95189
C	0.43894	4.00885	-0.35313
P	-0.08131	0.04004	-1.20162
C	-1.74597	-0.24621	-0.45741
C	-2.20113	0.36734	0.71497
C	-3.47030	0.07862	1.21523
C	-4.29623	-0.82983	0.55468
C	-3.85334	-1.44341	-0.61619
C	-2.58989	-1.14453	-1.12282
C	0.89740	-1.22740	-0.28105
C	0.37411	-2.15516	0.62722
C	1.18898	-3.11528	1.22760
C	2.55006	-3.16339	0.93360
C	3.10118	-2.25171	0.03454
C	2.26302	-1.31658	-0.54880
F	2.80058	-0.43098	-1.41426
H	1.37933	4.97061	1.32979
H	1.99100	2.83010	2.42895
H	1.38834	0.67694	1.38215
H	-0.40094	2.78491	-1.91005
H	0.17345	4.94062	-0.84285
H	-1.56379	1.07403	1.23900
H	-3.81293	0.56381	2.12413
H	-5.28337	-1.05350	0.94715
H	-4.49369	-2.14603	-1.14045
H	-2.25243	-1.61343	-2.04431
H	-0.68452	-2.12368	0.86736
H	0.75763	-3.82281	1.92795
H	3.18636	-3.90764	1.40123
H	4.15524	-2.25335	-0.21996

P_2F_C6H4_Ph2_H_gas
35

C	-0.45603	4.30097	0.67676
C	0.33626	4.04173	-0.44326
C	0.49088	2.73668	-0.89712
C	-0.15662	1.69541	-0.21951
C	-0.95193	1.94912	0.90231
C	-1.09711	3.26092	1.34765
P	0.10121	0.01335	-0.77967
C	1.73147	-0.58906	-0.33759
C	2.46946	0.05762	0.65921
C	3.71670	-0.45012	1.01693
C	4.21612	-1.58634	0.38310
C	3.47601	-2.22726	-0.61287
C	2.22932	-1.73223	-0.97788
C	-1.15482	-1.09299	-0.13911
C	-0.87965	-2.05262	0.84388
C	-1.90685	-2.86093	1.31961
C	-3.20163	-2.71784	0.81619
C	-3.49270	-1.76938	-0.16399
C	-2.45879	-0.97303	-0.61937
F	-2.69052	-0.04210	-1.56007
H	-0.57647	5.32139	1.02526
H	0.82869	4.85531	-0.96452
H	1.10864	2.53534	-1.76886
H	-1.45586	1.13787	1.42049
H	-1.71434	3.46902	2.21475
H	2.08142	0.94838	1.14539
H	4.29793	0.04549	1.78680
H	5.19002	-1.97456	0.66249
H	3.87189	-3.10805	-1.10653
H	1.65282	-2.23249	-1.75237
H	0.13151	-2.16415	1.22491
H	-1.69813	-3.60497	2.07962
H	-3.99761	-3.35397	1.18858
H	-4.48908	-1.64223	-0.57167
H	0.00453	0.00233	-2.17814

P_C2F5_3_gas
22

P	0.10314	-0.25771	-0.75866
C	-1.11633	-1.20821	0.37849
C	0.13319	1.45362	0.08868
C	1.66157	-0.98335	0.08020
C	-2.48460	-1.38071	-0.31347
F	-0.63918	-2.45260	0.59209
F	-1.32060	-0.62144	1.57060
C	-1.16120	2.28945	-0.02696
F	0.48881	1.43149	1.38447
F	1.08056	2.14177	-0.59994
F	1.80377	-2.21445	-0.46543
C	2.98244	-0.22830	-0.19936
F	1.55306	-1.12291	1.41410
F	-2.34403	-2.14323	-1.39724
F	-3.35502	-1.95019	0.50960
F	-2.95562	-0.18941	-0.69223
F	-2.09033	1.84538	0.81243
F	-0.89481	3.55812	0.26431
F	-1.63104	2.22239	-1.27514
F	3.03054	0.13902	-1.48298
F	3.07932	0.85143	0.56775
F	4.01262	-1.02752	0.05697

P_C2F5_3_H_gas
23

P	-0.07196	-0.07550	0.59536
C	0.74340	-1.41021	-0.52764
C	0.17303	1.70419	-0.06399
C	-1.93935	-0.42133	0.81710
C	2.01404	-1.94959	0.17508
F	-0.12776	-2.39378	-0.71162
F	1.04928	-0.83383	-1.68345
C	1.64926	2.16819	0.03243
F	-0.24734	1.74814	-1.32198
F	-0.59249	2.46774	0.71763
F	-2.35384	0.32929	1.83281
C	-2.74068	-0.10766	-0.47214
F	-2.03487	-1.71249	1.12688
F	2.66055	-2.76785	-0.61670
F	2.77712	-0.90264	0.49922
F	1.63588	-2.56663	1.29178
F	1.72206	3.45076	-0.23384
F	2.07651	1.92296	1.27327
F	2.37363	1.46628	-0.82788

F	-2.03135	-0.58482	-1.50351
F	-3.92028	-0.67648	-0.42455
F	-2.85103	1.20638	-0.59444
H	0.45662	-0.14642	1.89194

P_C3F7_3_gas
31

P	0.26735	-0.23048	0.82849
C	-0.10362	1.33610	-0.22314
C	-0.87701	-1.52087	-0.01254
C	1.81759	-0.78119	-0.16884
C	-1.01487	2.30388	0.56998
F	1.05393	1.98980	-0.45177
F	-0.67133	1.06801	-1.41459
C	-2.38971	-1.30319	0.21513
F	-0.65497	-1.64587	-1.33310
F	-0.56429	-2.69946	0.58325
F	1.85639	-0.29224	-1.42385
C	3.10607	-0.36190	0.57982
F	1.82639	-2.12743	-0.25028
F	-0.27446	2.87136	1.53591
F	-2.99544	-3.00239	-1.32344
F	-2.00554	1.59925	1.15192
C	-3.27961	-2.53001	-0.11250
F	-2.58750	-0.99889	1.51257
F	-2.80867	-0.28279	-0.54997
F	3.22102	-1.14269	1.66757
C	4.40769	-0.48350	-0.24561
F	2.99099	0.91789	0.98308
C	-1.66190	3.42489	-0.27476
F	-2.21375	4.32117	0.53858
F	-0.73704	4.02611	-1.02012
F	-2.60548	2.92375	-1.06489
F	4.48138	-1.68614	-0.81174
F	4.44802	0.44924	-1.19094
F	5.45162	-0.32023	0.56298
F	-3.10596	-3.49359	0.78543
F	-4.55398	-2.14690	-0.08580

P_C3F7_3_H_gas
32

P	-0.10443	-0.06639	0.64921
C	0.55885	1.37366	-0.44906
C	-1.62394	-0.94113	-0.13427
C	1.29554	-1.31255	1.03621
C	0.72386	2.62054	0.44865
F	1.72984	0.98674	-0.94828
F	-0.31572	1.58596	-1.42402
C	-2.89541	-0.23999	0.39497
F	-1.54302	-0.85757	-1.45571
F	-1.56384	-2.21314	0.25670
F	0.91353	-2.00561	2.10317
C	1.57419	-2.25136	-0.17894
F	2.35654	-0.56042	1.34082
F	1.47886	2.23594	1.49577
H	-0.48688	0.43086	1.90280
F	-0.50015	2.94516	0.89184
C	-4.20895	-0.64157	-0.32226
F	-2.98316	-0.51772	1.70266
F	-2.67661	1.08366	0.24518
F	1.05157	-1.62620	-1.26462
C	3.07708	-2.53249	-0.42878
F	0.92821	-3.39587	0.01263
C	1.37555	3.84662	-0.23965
F	2.62405	3.54987	-0.57092
F	0.68030	4.14842	-1.32909
F	1.35879	4.85973	0.60660
F	3.67318	-1.41768	-0.83368
F	3.62028	-2.94970	0.70940
F	3.18941	-3.46382	-1.35609
F	-5.22814	-0.12837	0.34052
F	-4.19165	-0.17703	-1.56265
F	-4.29198	-1.96738	-0.33827

P_C4F9_3_gas
40

P	-0.13303	-0.07651	-0.70884
C	1.06394	-1.34082	0.09962
C	-1.68481	-0.55565	0.31370
C	0.38149	1.42839	0.36583
C	1.24845	-2.56386	-0.82847
F	2.28009	-0.76587	0.23045
F	0.66388	-1.75717	1.31570
C	-2.96419	-0.01387	-0.37454
F	-1.78533	-1.89976	0.34262
F	-1.64892	-0.11025	1.58507
F	0.93566	1.08130	1.54421
C	1.37631	2.32592	-0.41454
F	-0.71373	2.17202	0.62121
F	1.94428	-2.15636	-1.90435
F	-4.24576	-1.16756	1.23933
F	0.03293	-2.97492	-1.24231
C	-4.21594	-0.02185	0.53725
F	-2.74154	1.25004	-0.78184
F	-3.19777	-0.77206	-1.46230
F	0.67980	2.98994	-1.35601
C	2.13468	3.34021	0.47628
F	2.28004	1.54249	-1.03310
C	1.94908	-3.81285	-0.22989
C	3.32957	-3.59480	0.42880
F	1.13737	-4.36793	0.68290
F	2.11886	-4.67649	-1.24633
F	1.27927	3.87663	1.36348
F	3.10598	2.68922	1.13334
C	2.78985	4.49868	-0.31475
F	-4.13491	1.01543	1.38356
C	-5.55217	0.09749	-0.23557
F	3.19828	-2.90981	1.56157
F	3.86515	-4.77971	0.70394
F	4.13798	-2.93275	-0.39554
F	3.48179	4.01215	-1.34438
F	1.86652	5.34023	-0.76722
F	3.61617	5.15963	0.48990
F	-5.48650	1.10468	-1.10563
F	-5.81891	-1.02823	-0.88900
F	-6.53313	0.33464	0.62966

P_C4F9_3_H_gas
41

P	0.28118	-0.08506	-0.80824
C	-0.13092	1.57017	0.08587
C	1.87356	-0.84195	-0.04404
C	-1.08325	-1.44377	-0.89493
C	-1.56680	2.03016	-0.26208
F	0.04230	1.40023	1.38712
F	0.76348	2.43769	-0.39808
C	3.06637	0.13844	-0.19513
F	1.63942	-1.11011	1.23371
F	2.09067	-1.96667	-0.73110
F	-0.62979	-2.33516	-1.77314
C	-1.30367	-2.07779	0.50185
F	-2.17638	-0.85180	-1.36523
F	-2.41328	1.22980	0.39776
F	4.34481	-1.40315	1.01721
F	-1.70633	1.82494	-1.58902
C	4.44379	-0.55245	-0.01597
F	2.97529	0.67315	-1.43023
F	2.89019	1.10755	0.71178
F	-1.18760	-1.05274	1.37876
C	-2.66675	-2.77099	0.77583
F	-0.29773	-2.93311	0.70685
C	-1.83168	3.52137	0.07478
C	-3.33968	3.87953	0.11950
F	-1.28021	3.77599	1.27148
F	-1.23454	4.26448	-0.86283
F	-2.59822	-3.24966	2.02028
F	-3.62146	-1.83397	0.69993
C	-3.01923	-3.92393	-0.18996
F	4.70573	-1.23370	-1.13675
C	5.60061	0.44433	0.25234
F	-3.92807	3.39467	-0.97363
F	-3.89957	3.34598	1.19672
F	-3.46781	5.19349	0.15247
F	-4.18117	-4.44411	0.14904
F	-3.09088	-3.42956	-1.43098
F	-2.07129	-4.85256	-0.15022
F	6.75095	-0.19563	0.14510
F	5.54150	1.42366	-0.64942
F	5.48276	0.95630	1.46982
H	0.54331	0.21318	-2.15147

P_C6F5_3_gas
34

C	3.23103	-2.61031	0.77531
C	3.17887	-2.53875	-0.60934
C	2.18248	-1.78108	-1.21622
C	1.22771	-1.08997	-0.47503
C	1.31202	-1.18511	0.91374
C	2.29177	-1.93082	1.54673
F	2.34211	-2.01050	2.87275
F	0.40833	-0.56302	1.67415
P	0.00035	-0.04609	-1.36907
C	-1.62113	-0.59146	-0.67425
C	-1.90913	-1.81598	-0.07816
C	-3.20400	-2.17841	0.27771
C	-4.25833	-1.31220	0.02833
C	-4.01122	-0.08667	-0.57975
C	-2.71194	0.24076	-0.92645
F	-2.49531	1.42981	-1.50334
F	-5.01325	0.75127	-0.82472
F	-5.49558	-1.65308	0.36264
F	-3.43491	-3.35711	0.84817
F	-0.94867	-2.71189	0.17342
C	0.32886	1.53890	-0.46150
C	-0.36103	2.10486	0.60964
C	-0.01189	3.34168	1.14015
C	1.06338	4.04353	0.61298
C	1.78542	3.50414	-0.44414
C	1.40702	2.27386	-0.95905
F	2.13027	1.77256	-1.96685
F	2.82392	4.16286	-0.94865
F	1.40434	5.21978	1.12094
F	-0.69745	3.85118	2.15897
F	-1.39229	1.48409	1.18360
F	2.16780	-1.73669	-2.54780
F	4.07828	-3.18661	-1.34263
F	4.17525	-3.32715	1.36735

P_C6F5_3_H_gas
35

C	2.18125	-3.84325	-0.31672
C	2.48518	-3.20284	0.88481
C	1.81612	-2.03433	1.21048
C	0.84287	-1.49365	0.36438
C	0.56300	-2.15042	-0.83697
C	1.21944	-3.32062	-1.18218
F	0.94781	-3.93358	-2.31818
F	-0.34047	-1.64505	-1.66927
P	-0.00154	0.00113	0.85981
C	0.86981	1.47744	0.35629
C	1.55093	1.56462	-0.86092
C	2.23949	2.71335	-1.21507
C	2.24302	3.80184	-0.34171
C	1.56467	3.74355	0.87592
C	0.88311	2.58467	1.21038
F	0.22584	2.52400	2.36582
F	1.57214	4.78038	1.69101
F	2.89066	4.89460	-0.67120
F	2.87979	2.78518	-2.36607
F	1.53445	0.53416	-1.69892
C	-1.71833	0.01621	0.36547
C	-2.13854	0.55296	-0.85450
C	-3.47880	0.57347	-1.20370
C	-4.42035	0.04067	-0.32200
C	-4.02667	-0.50736	0.89892
C	-2.68092	-0.51712	1.22818
F	-2.29614	-1.04655	2.38669
F	-4.92575	-1.01075	1.72212
F	-5.69172	0.05335	-0.64690
F	-3.86540	1.08221	-2.35766
F	-1.23976	1.04499	-1.69984
F	2.10557	-1.41121	2.35026
F	3.39919	-3.70413	1.69271
F	2.80944	-4.94963	-0.63833

H	0.00076	0.00608	2.25631

P_C6F5_Ph2_gas

34

C	2.51427	3.84545	0.76904
C	2.74823	2.60492	1.36207
C	2.24998	1.44164	0.77994
C	1.51761	1.50814	-0.41058
C	1.30362	2.75573	-1.00966
C	1.78811	3.92064	-0.41757
P	0.81917	0.05361	-1.31102
C	1.71032	-1.36645	-0.55361
C	1.07452	-2.46902	0.02313
C	1.81736	-3.56257	0.46784
C	3.20386	-3.56809	0.34221
C	3.84698	-2.47629	-0.24121
C	3.10536	-1.38956	-0.69417
C	-0.84386	-0.06573	-0.49545
C	-1.98495	-0.06795	-1.29705
C	-3.27136	-0.10620	-0.76955
C	-3.44189	-0.14256	0.60643
C	-2.33004	-0.14384	1.44098
C	-1.05980	-0.10544	0.88388
F	-0.02778	-0.12301	1.72888
F	-2.49538	-0.18504	2.76170
F	-4.66279	-0.17954	1.12685
F	-4.33481	-0.10570	-1.57063
F	-1.88122	-0.03116	-2.62722
H	2.90227	4.74938	1.22805
H	3.31529	2.54186	2.28564
H	2.42791	0.48174	1.25503
H	0.75884	2.81434	-1.94926
H	1.60996	4.88144	-0.89009
H	-0.00633	-2.48428	0.13169
H	1.30739	-4.40881	0.91745
H	3.78061	-4.41798	0.69262
H	4.92716	-2.47231	-0.34863
H	3.61721	-0.54652	-1.15338

P_C6F5_Ph2_H_gas

35

C	2.87091	3.91348	0.07800
C	3.63565	2.77214	-0.16484
C	3.01090	1.55889	-0.43363
C	1.61060	1.50322	-0.45887
C	0.83893	2.64567	-0.21744
C	1.47801	3.85179	0.05216
P	0.82364	-0.05109	-0.87734
C	1.72029	-1.46627	-0.25200
C	1.81647	-2.60749	-1.05903
C	2.48304	-3.72811	-0.57314
C	3.04670	-3.70335	0.70221
C	2.95148	-2.56186	1.50023
C	2.28750	-1.43459	1.02927
C	-0.90038	-0.03709	-0.36100
C	-1.94212	-0.19168	-1.27724
C	-3.26832	-0.20356	-0.86768
C	-3.56452	-0.06111	0.48588
C	-2.54343	0.09416	1.42368
C	-1.22854	0.10270	0.98905
F	-0.25016	0.24907	1.88172
F	-2.83379	0.22908	2.70536
F	-4.81775	-0.07258	0.88441
F	-4.24227	-0.34996	-1.74782
F	-1.67554	-0.33038	-2.57434
H	3.36380	4.85633	0.29056
H	4.71857	2.82486	-0.14195
H	3.60764	0.66919	-0.61475
H	-0.24622	2.60147	-0.23677
H	0.88802	4.74115	0.24419
H	1.38275	-2.62591	-2.05542
H	2.56655	-4.61501	-1.19150
H	3.56825	-4.57825	1.07605
H	3.39591	-2.54917	2.48938
H	2.21613	-0.54397	1.64694
H	0.74065	-0.22306	-2.26437

P_CF3_2Me_gas

13

P	-0.00063	0.66198	-0.85546
C	-0.00610	2.14434	0.24419
C	-1.40912	-0.27095	-0.00739
C	1.41214	-0.26685	-0.00712
H	-0.86677	2.76311	-0.01830
H	0.90116	2.72048	0.04698
H	-0.04834	1.87919	1.30176
F	-2.55066	0.40926	-0.23187
F	-1.28871	-0.40447	1.32157
F	-1.55876	-1.49782	-0.52156
F	2.57608	0.24229	-0.45518
F	1.38811	-1.56952	-0.31808
F	1.43858	-0.17216	1.32993

P_CF3_2Me_H_gas

14

P	-0.00010	0.65430	-0.54374
C	-0.00025	2.29735	0.17836
C	-1.52341	-0.31774	0.01726
C	1.52352	-0.31761	0.01723
H	-0.89623	2.83275	-0.14860
H	0.89570	2.83290	-0.14843
H	-0.00036	2.19753	1.26801
F	-2.59691	0.34505	-0.38793
F	-1.50766	-0.39228	1.33798
F	-1.49968	-1.52473	-0.51381
F	2.59692	0.34521	-0.38811
F	1.49982	-1.52466	-0.51368
F	1.50789	-0.39200	1.33796
H	-0.00017	0.64118	-1.94369

P_CF3_3_gas

13

C	0.00000	1.64923	0.01600
P	0.00000	0.00000	0.95707
C	-1.42827	-0.82461	0.01600

F	-1.48658	-2.11102	0.38527
F	-2.57149	-0.23191	0.38527
F	-1.35796	-0.78402	-1.31291
C	1.42827	-0.82461	0.01600
F	1.48658	-2.11102	0.38527
F	1.35796	-0.78402	-1.31291
F	2.57149	-0.23191	0.38527
F	1.08491	2.34293	0.38527
F	0.00000	1.56804	-1.31291
F	-1.08491	2.34293	0.38527

P_CF3_3_H_gas
14

C	0.80371	-1.60539	-0.00282
P	0.00058	0.00120	0.64786
C	0.98827	1.49842	-0.00963
F	0.64991	2.55721	0.69603
F	2.27088	1.22902	0.12946
F	0.67630	1.66039	-1.27895
C	-1.79245	0.10630	-0.00285
F	-2.20046	1.35186	0.13559
F	-1.78160	-0.24715	-1.27157
F	-2.53706	-0.71521	0.70713
H	0.00254	0.00426	2.04908
F	-0.06857	-2.58231	0.14518
F	1.09511	-1.42330	-1.27429
F	1.89283	-1.83530	0.70027

P_CF3Me2_gas
13

P	0.89983	0.69737	0.00000
C	0.05944	1.53665	1.41964
C	0.05944	1.53665	-1.41964
C	-0.18557	-0.84139	0.00000
H	0.45797	2.55117	1.50112
H	0.29168	1.00849	2.34728
H	-1.02467	1.58619	1.29024
H	0.45797	2.55117	-1.50112
H	-1.02467	1.58619	-1.29024
H	0.29168	1.00849	-2.34728
F	0.05944	-1.60051	1.08524
F	0.05944	-1.60051	-1.08524
F	-1.51302	-0.59272	0.00000

P_CF3Me2_H_gas
14

P	-0.60999	0.74685	0.00000
C	-0.13044	1.59295	-1.51225
C	-0.13044	1.59295	1.51225
C	0.22495	-0.93600	0.00000
H	-0.62738	2.56524	-1.55986
H	-0.42104	0.98969	-2.37605
H	0.95368	1.73548	-1.50924
H	-0.62738	2.56524	1.55986
H	0.95368	1.73548	1.50924
H	-0.42104	0.98969	2.37605
F	-0.13044	-1.60935	-1.08596
F	-0.13044	-1.60935	1.08596
F	1.54234	-0.75233	0.00000
H	-1.97824	0.45630	0.00000

PF3_gas
4

P	0.00005	-0.00001	-0.51491
F	-0.44858	1.30176	0.28605
F	1.35179	-0.26248	0.28610
F	-0.90329	-1.03927	0.28604

PF3_H_gas
5

P	0.00007	-0.00010	0.30642
F	-0.34248	1.37054	-0.23283
F	-1.01599	-0.98165	-0.23292
F	1.35832	-0.38869	-0.23300
H	0.00037	-0.00033	1.69233

PMe3_gas
13

P	0.00002	0.00005	-0.61412
C	-0.93965	-1.32460	0.28361
C	1.61701	-0.15142	0.28365
C	-0.67739	1.47595	0.28365
H	-0.55413	-2.30728	-0.00162
H	-1.99471	-1.28483	-0.00083
H	-0.86124	-1.21490	1.37115
H	2.11033	-1.08479	-0.00118
H	1.48283	-0.13882	1.37120
H	2.27514	0.67402	-0.00126
H	-1.72134	1.63303	-0.00119
H	-0.11584	2.36997	-0.00108
H	-0.62119	1.35329	1.37117

PMe3_H_gas
14

P	0.00003	-0.00004	0.34344
C	-1.17240	-1.25093	-0.21609
C	-0.49722	1.64078	-0.21607
C	1.66958	-0.38978	-0.21610
H	-2.17278	-1.01557	0.15413
H	-0.87244	-2.23407	0.15358
H	-1.18672	-1.26577	-1.30901
H	-1.49865	1.87245	0.15365
H	-0.50292	1.66059	-1.30899
H	0.20676	2.38950	0.15414
H	1.96581	-1.37402	0.15365
H	2.37102	0.36134	0.15405
H	1.68970	-0.39425	-1.30902
H	0.00002	-0.00005	1.74176

Cartesian coordinates for solvent-phase structures (SMD model)

P_26_F2_C6H3_2Ph_solvent
34

C	-0.27083	4.28875	-0.90251
C	-1.03243	3.24367	-1.42765
C	-0.97958	1.97501	-0.85079
C	-0.16925	1.74339	0.26540
C	0.58249	2.79940	0.79664
C	0.53981	4.06409	0.20995
P	0.00443	0.12299	1.14136
C	-1.38266	-0.86114	0.42717
C	-2.67950	-0.39762	0.66632
C	-3.83758	-1.04487	0.27659
C	-3.71157	-2.26505	-0.38536
C	-2.45154	-2.80421	-0.62910
C	-1.33152	-2.09915	-0.21412
F	-0.12898	-2.67100	-0.44167
F	-2.79956	0.77566	1.33262
C	1.50795	-0.50576	0.27983
C	1.65865	-0.63240	-1.10276
C	2.83002	-1.02358	-1.72427
C	3.93621	-1.30889	-0.92199
C	3.85557	-1.20556	0.46327
C	2.64859	-0.80853	1.02112
F	2.58504	-0.70974	2.36752
F	0.58323	-0.37818	-1.87678
H	-0.31309	5.27450	-1.35601
H	-1.66700	3.41318	-2.29259
H	-1.56912	1.16903	-1.27959
H	1.20509	2.63466	1.67388
H	1.13103	4.87283	0.62898
H	-4.80196	-0.60022	0.49608
H	-4.59907	-2.80174	-0.70302
H	-2.32044	-3.75794	-1.12859
H	2.86581	-1.10485	-2.80504
H	4.86818	-1.61684	-1.38424
H	4.69955	-1.42334	1.10823

P_26_F2_C6H3_Ph2_solvent
34

C	-3.88658	-0.82932	1.11388
C	-2.74869	-0.50126	1.85047
C	-1.56831	-0.27509	1.16358
C	-1.43980	-0.34963	-0.22628
C	-2.61593	-0.68574	-0.89803
C	-3.83055	-0.92688	-0.27379
C	0.61678	1.60789	-0.42307
C	1.57631	1.74196	0.58664
C	1.92361	3.00578	1.06395
C	1.31033	4.14756	0.54614
C	0.35118	4.02242	-0.45908
C	0.01628	2.76013	-0.94859
C	1.29548	-1.20592	-0.48437
C	2.63385	-1.07469	-0.88767
C	3.59480	-1.99236	-0.47056
C	3.22840	-3.07089	0.33757
C	1.89800	-3.22133	0.72388
C	0.93592	-2.29389	0.31731
H	-4.82423	-1.01246	1.62808
H	-2.76488	-0.42424	2.93208
F	-2.57795	-0.78475	-2.24542
H	-4.69983	-1.18215	-0.86958
H	2.67076	3.09677	1.84709
H	1.58204	5.12969	0.92150
H	-0.12721	4.90552	-0.87217
H	4.62761	-1.86959	-0.78302
H	3.97522	-3.79080	0.65854
H	1.60224	-4.06043	1.34676
F	-0.46863	0.02247	1.88630
P	0.09762	0.00402	-1.18232
H	-0.71809	2.67098	-1.74681
H	2.05077	0.86147	1.01061
H	-0.09548	-2.43165	0.62962
H	2.92942	-0.24545	-1.52771

P_26F_C6H3_2Ph_H_solvent
35

C	-1.30554	4.41851	0.01934
C	-2.08611	3.57524	-0.77522
C	-1.67905	2.26644	-1.01022
C	-0.48251	1.81409	-0.44002
C	0.30663	2.65000	0.35323
C	-0.11488	3.95938	0.58004
P	0.02224	0.12397	-0.77807
C	1.62400	-0.26161	-0.07501
C	2.78054	-0.29415	-0.85705
C	4.02254	-0.61320	-0.34062
C	4.11418	-0.90994	1.01855
C	2.99202	-0.88791	1.84747
C	1.77630	-0.56203	1.28039
F	0.67419	-0.51812	2.04905
F	2.66890	0.00117	-2.16280
C	-1.20570	-1.07019	-0.24197
C	-1.34626	-2.27430	-0.93710
C	-2.28005	-3.23527	-0.60283
C	-3.11373	-2.98439	0.48659
C	-3.01182	-1.80504	1.22314
C	-2.05912	-0.87628	0.84724
F	-1.93983	0.25633	1.55594
F	-0.51914	-2.49229	-1.97520
H	-1.62823	5.43930	0.19938
H	-3.01112	3.93644	-1.21250
H	-2.28611	1.60619	-1.62454
H	1.23509	2.29400	0.79036
H	0.48997	4.61700	1.19565
H	4.88819	-0.62407	-0.99304
H	5.08071	-1.16106	1.44229
H	3.05070	-1.11242	2.90632
H	-2.34247	-4.14975	-1.18140
H	-3.85422	-3.72472	0.76977

H -3.64872 -1.60104 2.07632
H 0.14699 -0.05927 -2.15619

P_26F_C6H3_Ph2_H_solvent
35

C -4.08087 -0.47247 0.92598
C -2.98975 -0.34683 1.78700
C -1.73845 -0.20106 1.22310
C -1.51598 -0.17470 -0.15510
C -2.64347 -0.30203 -0.96830
C -3.92075 -0.45076 -0.45829
C 0.83566 1.59457 -0.40048
C 2.20087 1.81407 -0.62598
C 2.74148 3.06574 -0.34760
C 1.92746 4.08454 0.15144
C 0.56925 3.85907 0.37159
C 0.01395 2.61108 0.09552
C 1.20212 -1.36443 -0.29267
C 1.97174 -1.27029 0.87187
C 2.75383 -2.35684 1.25443
C 2.76355 -3.51988 0.48254
C 1.99543 -3.60527 -0.67921
C 1.21120 -2.52489 -1.07527
H -5.07555 -0.58880 1.34290
H -3.09920 -0.35960 2.86533
F -2.47524 -0.27924 -2.30154
H -4.76044 -0.54625 -1.13725
H 3.79811 3.24386 -0.51868
H 2.35521 5.05826 0.36953
H -0.06173 4.65214 0.75934
H 3.35649 -2.29334 2.15456
H 3.37699 -4.36261 0.78621
H 2.01051 -4.50812 -1.28089
F -0.66053 -0.06948 2.01854
P 0.14465 -0.01129 -0.81962
H -1.04557 2.43960 0.26425
H 2.83528 1.02063 -1.01224
H 0.61541 -2.58284 -1.98216
H 1.96808 -0.36363 1.46997
H -0.00513 -0.13915 -2.20300

P_2F_C6H4_2Ph_H_solvent
35

C 2.97648 -3.35500 0.52460
C 2.31586 -3.43617 -0.70241
C 1.42516 -2.43518 -1.08067
C 1.20609 -1.35702 -0.21580
C 1.86589 -1.26629 1.01431
C 2.75356 -2.27529 1.38037
P 0.02031 -0.10132 -0.69687
C -1.63099 -0.54863 -0.15188
C -2.73170 0.10237 -0.70525
C -4.03139 -0.18215 -0.33260
C -4.23074 -1.15927 0.64294
C -3.14981 -1.82775 1.22010
C -1.84930 -1.52706 0.82475
C 0.46639 1.50333 -0.02444
C -0.24018 2.10314 1.02403
C 0.17839 3.33462 1.52051
C 1.29631 3.96778 0.97474
C 2.01284 3.38354 -0.07001
C 1.58024 2.15983 -0.54311
F 2.25224 1.56017 -1.54855
H 3.67223 -4.13657 0.81388
H 2.49702 -4.27439 -1.36727
H 0.90973 -2.48898 -2.03566
H 1.69111 -0.42270 1.67693
H 3.27230 -2.21548 2.33151
F -2.50140 1.04714 -1.64092
H -4.85476 0.35004 -0.79642
H -5.24319 -1.39863 0.95183
H -3.31843 -2.58622 1.97654
H -1.00643 -2.04947 1.26779
H -1.11045 1.60726 1.44503
H -0.36891 3.79971 2.33294
H 1.61807 4.92835 1.36397
H 2.88396 3.85632 -0.51033
H 0.01684 -0.02393 -2.09349

P_2F_C6H4_2Ph_solvent
34

C -2.90095 -3.24741 0.70707
C -2.35681 -3.40199 -0.56766
C -1.50740 -2.42352 -1.08600
C -1.18422 -1.29075 -0.32898
C -1.73458 -1.14246 0.95042
C -2.59130 -2.11547 1.46376
P -0.02145 -0.08082 -1.08629
C -0.43245 1.45186 -0.14414
C -1.60394 2.12376 -0.48744
C -2.02379 3.30217 0.10387
C -1.22153 3.85631 1.10142
C -0.03857 3.22157 1.47910
C 0.34844 2.03055 0.86355
C 1.56498 -0.56266 -0.27933
C 1.73185 -1.64713 0.59096
C 2.99253 -1.98300 1.08651
C 4.11336 -1.23707 0.72296
C 3.97972 -0.15127 -0.14269
C 2.71527 0.14693 -0.61852
F 2.57910 1.20873 -1.45469
H -3.56774 -4.00420 1.10956
H -2.59820 -4.27791 -1.16239
H -1.09278 -2.54274 -2.08450
H -1.49159 -0.26931 1.55108
H -3.01419 -1.99118 2.45633
F -2.38767 1.57813 -1.45344
H -2.95240 3.76424 -0.21494
H -1.52534 4.78137 1.58102
H 0.58461 3.64948 2.25783
H 1.26784 1.54445 1.17759
H 0.86793 -2.23708 0.88272
H 3.09578 -2.82795 1.75976
H 5.09380 -1.49556 1.11019
H 4.82737 0.45448 -0.44599

P_2F_C6H4_Ph2_H_solvent

35

C	-1.50865	4.04802	0.69983
C	-0.78034	3.98758	-0.48991
C	-0.30148	2.76498	-0.95206
C	-0.56060	1.60610	-0.21027
C	-1.28936	1.65886	0.98205
C	-1.76274	2.88928	1.43366
P	0.11404	0.04822	-0.79622
C	1.83588	-0.14884	-0.33227
C	2.31061	0.42396	0.85189
C	3.63936	0.22017	1.21734
C	4.47764	-0.54554	0.40593
C	3.99660	-1.11295	-0.77543
C	2.67034	-0.91777	-1.15189
C	-0.84715	-1.33604	-0.17122
C	-0.31886	-2.26566	0.73126
C	-1.11670	-3.30890	1.19431
C	-2.43729	-3.42842	0.75901
C	-2.98041	-2.51170	-0.14186
C	-2.16918	-1.48490	-0.58482
F	-2.66160	-0.57723	-1.45493
H	-1.88263	5.00353	1.05454
H	-0.58831	4.89062	-1.06024
H	0.26410	2.71342	-1.87883
H	-1.48850	0.75376	1.55021
H	-2.33135	2.93978	2.35649
H	1.65468	1.02215	1.47900
H	4.01892	0.66184	2.13294
H	5.51328	-0.69812	0.69387
H	4.65349	-1.70382	-1.40545
H	2.28926	-1.35626	-2.07038
H	0.70994	-2.17115	1.06670
H	-0.70608	-4.02843	1.89412
H	-3.05544	-4.24357	1.12132
H	-4.00308	-2.58385	-0.49589
H	0.04956	0.04260	-2.19405

P_2F_C6H4_Ph2_solvent
34

C	1.28596	3.99056	0.84681
C	1.57976	2.78003	1.47539
C	1.18493	1.57342	0.89618
C	0.49796	1.56571	-0.32387
C	0.21774	2.78577	-0.95417
C	0.60099	3.99214	-0.36883
P	-0.09081	0.04008	-1.17562
C	-1.76703	-0.18779	-0.44382
C	-2.19175	0.42376	0.74184
C	-3.47534	0.18827	1.23558
C	-4.34436	-0.66503	0.55435
C	-3.92958	-1.27787	-0.62850
C	-2.65069	-1.03280	-1.12874
C	0.85229	-1.26721	-0.27780
C	0.29946	-2.20814	0.59986
C	1.08925	-3.20260	1.17944
C	2.45248	-3.27370	0.89492
C	3.03317	-2.35033	0.02476
C	2.21684	-1.38406	-0.53537
F	2.78092	-0.48118	-1.38002
H	1.59300	4.92816	1.30062
H	2.11359	2.77271	2.42121
H	1.41011	0.63886	1.40393
H	-0.30474	2.79317	-1.90840
H	0.37331	4.93002	-0.86677
H	-1.52259	1.08518	1.28565
H	-3.79478	0.67065	2.15478
H	-5.34264	-0.84727	0.94115
H	-4.60250	-1.93892	-1.16660
H	-2.33609	-1.50474	-2.05718
H	-0.76043	-2.16347	0.83348
H	0.63651	-3.91959	1.85692
H	3.06841	-4.04451	1.34717
H	4.09020	-2.37158	-0.21963

P_C2F5_3_H_solvent
23

P	-0.05498	-0.07742	0.70610
C	0.81412	-1.35740	-0.43034
C	0.06580	1.67713	-0.02914
C	-1.91460	-0.51052	0.82937
C	2.15085	-1.89019	0.15535
F	-0.01596	-2.38240	-0.61343
F	1.05396	-0.75118	-1.59458
C	1.47637	2.31885	0.04216
F	-0.31204	1.61021	-1.30800
F	-0.80395	2.41844	0.66095
F	-2.42903	0.24859	1.79310
C	-2.67557	-0.26433	-0.50176
F	-1.98813	-1.79635	1.16529
F	2.75143	-2.61975	-0.76875
F	2.92429	-0.86044	0.48756
F	1.91143	-2.63015	1.22895
F	1.39820	3.55487	-0.41830
F	1.91629	2.33097	1.29327
F	2.30344	1.60402	-0.71480
F	-1.89253	-0.65970	-1.51130
F	-3.80391	-0.94224	-0.51743
F	-2.91582	1.03368	-0.62779
H	0.44370	-0.10696	2.02211

P_C2F5_3_solvent
22

P	0.10474	-0.26175	-0.78802
C	-1.11900	-1.20332	0.35466
C	0.14787	1.44354	0.07722
C	1.65340	-0.99452	0.06745
C	-2.50536	-1.35690	-0.30595
F	-0.65382	-2.45554	0.55640
F	-1.29776	-0.62552	1.55844
C	-1.14102	2.29168	-0.02689
F	0.49906	1.40784	1.37729
F	1.10721	2.13181	-0.59404
F	1.80084	-2.23077	-0.46200
C	2.98018	-0.24006	-0.19514
F	1.52987	-1.12897	1.40413
F	-2.39702	-2.06994	-1.42516

F	-3.34139	-1.97421	0.52195
F	-3.00100	-0.15575	-0.60960
F	-2.04988	1.87027	0.84713
F	-0.85580	3.56393	0.23528
F	-1.64785	2.20816	-1.25774
F	3.02961	0.17579	-1.46181
F	3.08488	0.81188	0.61152
F	4.00775	-1.05302	0.03067

P_C3F7_3_H_solvent
32

P	-0.13123	-0.01978	0.75436
C	0.58850	1.37171	-0.36837
C	-1.73990	-0.68899	-0.06717
C	1.20354	-1.38369	1.01368
C	1.22439	2.49720	0.48808
F	1.50343	0.81561	-1.16440
F	-0.41046	1.86596	-1.09753
C	-2.98090	0.05386	0.49631
F	-1.65460	-0.48489	-1.38281
F	-1.82596	-1.99429	0.17870
F	0.97502	-1.96631	2.18269
C	1.21926	-2.44360	-0.13058
F	2.36841	-0.72552	1.06174
F	2.10362	1.92222	1.32419
H	-0.42473	0.47778	2.03794
F	0.24670	3.07796	1.19553
C	-4.25114	-0.11389	-0.38064
F	-3.23957	-0.40907	1.72146
F	-2.66276	1.35973	0.56709
F	0.68403	-1.85945	-1.22427
C	2.63905	-2.96214	-0.48168
F	0.45955	-3.47644	0.22942
C	1.96355	3.59540	-0.32007
F	2.97737	3.06916	-0.99601
F	1.12307	4.18303	-1.16378
F	2.43047	4.49685	0.53557
F	3.32230	-2.02849	-1.13096
F	3.27901	-3.28283	0.64056
F	2.52863	-4.03836	-1.24736
F	-5.30486	0.31139	0.30199
F	-4.13653	0.59273	-1.49740
F	-4.41186	-1.39969	-0.68450

P_C3F7_3_solvent
31

P	0.26760	-0.23374	0.88011
C	-0.08887	1.33918	-0.16916
C	-0.87284	-1.50911	0.00639
C	1.81318	-0.77673	-0.13325
C	-1.03164	2.30816	0.58801
F	1.07228	1.99826	-0.36715
F	-0.62111	1.07272	-1.38105
C	-2.39040	-1.29587	0.20999
F	-0.63486	-1.61504	-1.31649
F	-0.57192	-2.70128	0.58037
F	1.83820	-0.27439	-1.38699
C	3.12055	-0.37367	0.59417
F	1.81950	-2.12247	-0.23734
F	-0.32578	2.91573	1.55355
F	-2.91956	-3.04381	-1.30839
F	-2.02161	1.59749	1.16283
C	-3.26384	-2.53345	-0.12838
F	-2.61628	-0.98550	1.49981
F	-2.79918	-0.28058	-0.56832
F	3.27732	-1.19105	1.64828
C	4.39373	-0.46153	-0.28133
F	3.01473	0.89035	1.04349
C	-1.67799	3.39636	-0.30272
F	-2.23147	4.32668	0.46955
F	-0.75108	3.96574	-1.07140
F	-2.62015	2.86435	-1.07563
F	4.43486	-1.63143	-0.91719
F	4.41270	0.52153	-1.17617
F	5.46774	-0.35371	0.49617
F	-3.13257	-3.47011	0.80586
F	-4.53900	-2.15613	-0.17646

P_C4F9_3_H_solvent
41

P	0.30967	-0.03996	-0.97860
C	-0.20453	1.58059	-0.07583
C	1.89003	-0.71543	-0.12748
C	-0.98423	-1.46262	-0.96433
C	-1.69270	1.94558	-0.31584
F	0.04614	1.43265	1.22171
F	0.57782	2.53551	-0.58631
C	3.11637	0.21159	-0.32866
F	1.62152	-0.84002	1.17303
F	2.12104	-1.91641	-0.66271
F	-0.52596	-2.37290	-1.81800
C	-1.16061	-2.06361	0.45796
F	-2.12761	-0.94507	-1.41355
F	-2.44461	1.08784	0.38965
F	4.25881	-1.02328	1.33265
F	-1.94253	1.77581	-1.62422
C	4.45089	-0.42239	0.14892
F	3.21858	0.50056	-1.63264
F	2.86727	1.33754	0.36012
F	-1.08033	-1.02133	1.31336
C	-2.49710	-2.79101	0.77896
F	-0.13184	-2.88669	0.68403
C	-2.03067	3.40789	0.08426
C	-3.55200	3.67087	0.23112
F	-1.43307	3.68755	1.25285
F	-1.55449	4.22467	-0.86413
F	-2.39643	-3.21986	2.04424
F	-3.48775	-1.89222	0.69150
C	-2.85290	-3.99986	-0.11509
F	4.82441	-1.33534	-0.75794
C	5.59886	0.60841	0.30762
F	-4.19189	3.21095	-0.84228
F	-4.02642	3.07070	1.31627
F	-3.75218	4.97874	0.33452
F	-4.03587	-4.47488	0.24580
F	-2.90609	-3.60376	-1.38775
F	-1.93636	-4.95331	0.00916

F	6.74006	-0.04636	0.48370
F	5.68922	1.36079	-0.78674
F	5.37610	1.38761	1.35958
H	0.55886	0.24390	-2.33386

P_C4F9_3_solvent
40

P	-0.15061	-0.06714	-0.78665
C	1.00124	-1.36182	0.04340
C	-1.70571	-0.48393	0.26186
C	0.41805	1.41871	0.29095
C	1.14399	-2.62555	-0.83833
F	2.24049	-0.83064	0.15423
F	0.59713	-1.72674	1.27802
C	-2.98535	0.09101	-0.39920
F	-1.84980	-1.82437	0.31111
F	-1.63486	-0.03007	1.53227
F	0.92745	1.04535	1.48516
C	1.47276	2.28195	-0.45234
F	-0.64826	2.21086	0.52922
F	1.85352	-2.28735	-1.92875
F	-4.23902	-0.99794	1.28608
F	-0.08057	-3.02206	-1.23468
C	-4.20993	0.12731	0.55110
F	-2.73937	1.34551	-0.81943
F	-3.27465	-0.66630	-1.47284
F	0.83518	2.99771	-1.39610
C	2.25374	3.24144	0.48158
F	2.35973	1.47348	-1.06111
C	1.81311	-3.86754	-0.18668
C	3.20096	-3.65604	0.46209
F	0.98931	-4.36539	0.74875
F	1.95693	-4.77763	-1.16617
F	1.39977	3.80580	1.35322
F	3.16767	2.52930	1.15908
C	2.99730	4.37632	-0.26573
F	-4.08334	1.18373	1.36883
C	-5.56673	0.25006	-0.18555
F	3.08227	-2.94290	1.57956
F	3.72233	-4.84076	0.76241
F	4.01541	-3.02191	-0.37825
F	3.70613	3.86953	-1.27359
F	2.13555	5.26854	-0.74307
F	3.82173	4.98570	0.58086
F	-5.50464	1.21850	-1.09850
F	-5.87806	-0.89499	-0.78434
F	-6.51597	0.54566	0.69701

P_C6F5_3_solvent
34

C	3.27857	-2.57867	0.74926
C	3.22270	-2.48635	-0.63166
C	2.20850	-1.74612	-1.22514
C	1.23780	-1.08883	-0.47635
C	1.32845	-1.20211	0.90940
C	2.32535	-1.93380	1.52870
F	2.37877	-2.03108	2.85708
F	0.41390	-0.60897	1.68281
P	0.00010	-0.05087	-1.36076
C	-1.62103	-0.60451	-0.67355
C	-1.90571	-1.82060	-0.06065
C	-3.19997	-2.18708	0.28707
C	-4.25810	-1.33590	0.01653
C	-4.01461	-0.11955	-0.60548
C	-2.71638	0.21501	-0.94320
F	-2.50780	1.40127	-1.53349
F	-5.02348	0.70939	-0.87271
F	-5.49870	-1.68351	0.34457
F	-3.42641	-3.36188	0.87605
F	-0.94253	-2.70552	0.22172
C	0.31229	1.53669	-0.45403
C	-0.36452	2.07566	0.63796
C	-0.02745	3.30905	1.17935
C	1.02106	4.03846	0.64226
C	1.72573	3.53029	-0.43853
C	1.36107	2.30266	-0.96459
F	2.07084	1.83938	-2.00271
F	2.73987	4.22026	-0.96006
F	1.35184	5.21591	1.16225
F	-0.70252	3.79022	2.22391
F	-1.37580	1.42836	1.22431
F	2.19719	-1.68085	-2.55983
F	4.13922	-3.10167	-1.37870
F	4.24295	-3.28275	1.33118

P_C6F5_Ph2_H_solvent
35

C	2.84291	3.93874	0.29452
C	3.61789	2.83309	-0.05949
C	3.00504	1.63015	-0.39767
C	1.60665	1.54906	-0.37726
C	0.82305	2.65426	-0.02920
C	1.45117	3.85080	0.30844
P	0.83542	0.00702	-0.88319
C	1.73023	-1.44159	-0.32096
C	1.76091	-2.55406	-1.17098
C	2.42638	-3.70504	-0.75719
C	3.05528	-3.73710	0.48768
C	3.02533	-2.62148	1.32667
C	2.36137	-1.46492	0.92825
C	-0.88972	-0.02910	-0.35388
C	-1.93175	-0.01791	-1.27895
C	-3.25752	-0.05772	-0.87602
C	-3.55923	-0.11145	0.47717
C	-2.54079	-0.12545	1.42234
C	-1.22467	-0.08586	0.99810
F	-0.25798	-0.09925	1.91649
F	-2.83628	-0.17730	2.71760
F	-4.82336	-0.15012	0.87096
F	-4.23658	-0.04456	-1.77597
F	-1.68002	0.03555	-2.58621
H	3.32718	4.87303	0.56139
H	4.70052	2.90437	-0.06977
H	3.60934	0.76970	-0.67158
H	-0.26140	2.58953	-0.01756
H	0.85065	4.71150	0.58392
H	1.27530	-2.52113	-2.14245

H	2.45797	-4.57128	-1.40986
H	3.57715	-4.63483	0.80458
H	3.52077	-2.64949	2.29154
H	2.34423	-0.59467	1.57805
H	0.75975	-0.08724	-2.27572

P_C6F5_Ph2_solvent
34

C	2.53878	3.86550	0.69285
C	2.84026	2.62995	1.26798
C	2.33477	1.45374	0.71471
C	1.52892	1.50371	-0.42844
C	1.24238	2.74649	-1.00940
C	1.73460	3.92314	-0.44532
P	0.82626	0.03089	-1.29356
C	1.70347	-1.38986	-0.52288
C	1.05871	-2.45043	0.12171
C	1.79140	-3.54698	0.58088
C	3.17254	-3.59584	0.40168
C	3.82179	-2.54622	-0.25156
C	3.09143	-1.45685	-0.71985
C	-0.84016	-0.06035	-0.48935
C	-1.97603	-0.10966	-1.29326
C	-3.26305	-0.14284	-0.77324
C	-3.44218	-0.12502	0.59994
C	-2.33652	-0.07725	1.43737
C	-1.06506	-0.04466	0.88735
F	-0.03532	-0.01230	1.74015
F	-2.50872	-0.06494	2.76102
F	-4.66798	-0.15681	1.11555
F	-4.32287	-0.19062	-1.58313
F	-1.86673	-0.12912	-2.62779
H	2.93366	4.77879	1.12781
H	3.46678	2.58026	2.15371
H	2.56783	0.50016	1.17975
H	0.63261	2.79531	-1.90925
H	1.50058	4.87974	-0.90285
H	-0.01774	-2.43623	0.26958
H	1.27685	-4.36145	1.08204
H	3.74025	-4.44788	0.76319
H	4.89668	-2.57755	-0.40271
H	3.60682	-0.64995	-1.23700

P_CF3_2Me_H_solvent
14

P	0.00049	0.70439	-0.54558
C	0.00896	2.32205	0.20120
C	-1.49448	-0.30302	0.01156
C	1.49029	-0.30826	0.01156
H	-0.87493	2.85716	-0.15786
H	0.92180	2.83142	-0.12126
H	-0.01488	2.20649	1.28799
F	-2.60869	0.33591	-0.32672
F	-1.46759	-0.46529	1.33090
F	-1.48246	-1.49764	-0.57166
F	2.60129	0.29629	-0.39485
F	1.44168	-1.52952	-0.51120
F	1.50796	-0.40537	1.33726
H	0.00232	0.66504	-1.94470

P_CF3_2Me_solvent
13

P	0.00008	0.70394	-0.84528
C	-0.00676	2.17559	0.25339
C	-1.39403	-0.26674	-0.01176
C	1.39803	-0.26157	-0.01112
H	-0.82005	2.82936	-0.07093
H	0.93783	2.70610	0.10126
H	-0.12461	1.92715	1.30984
F	-2.53477	0.44292	-0.10221
F	-1.21134	-0.54314	1.29215
F	-1.60891	-1.44266	-0.62801
F	2.57671	0.13871	-0.52529
F	1.28607	-1.58169	-0.25101
F	1.49470	-0.11474	1.32058

P_CF3_3_solvent
13

C	-0.02041	1.64601	0.00000
P	-0.97348	0.00067	0.00000
C	-0.02053	-0.82262	-1.42435
F	-0.36779	-2.11601	-1.47777
F	-0.38950	-0.24637	-2.57617
F	1.31226	-0.76764	-1.35635
C	-0.02053	-0.82262	1.42435
F	-0.36779	-2.11601	1.47777
F	1.31226	-0.76764	1.35635
F	-0.38950	-0.24637	2.57617
F	-0.37929	2.34849	1.08370
F	1.31209	1.56142	0.00000
F	-0.37929	2.34849	-1.08370

P_CF3Me2_H_solvent
14

P	-0.61299	0.75855	0.00000
C	-0.12951	1.61015	-1.49656
C	-0.12951	1.61015	1.49656
C	0.22096	-0.92170	0.00000
H	-0.64958	2.57159	-1.50886
H	-0.41650	1.01621	-2.36683
H	0.95114	1.77157	-1.47364
H	-0.64958	2.57159	1.50886
H	0.95114	1.77157	1.47364
H	-0.41650	1.01621	2.36683
F	-0.12951	-1.62204	-1.08202
F	-0.12951	-1.62204	1.08202
F	1.55041	-0.79117	0.00000
H	-1.96949	0.42864	0.00000

P_CF3Me2_solvent
13

P	0.88547	0.70910	0.00000
C	0.06569	1.54833	1.42174
C	0.06569	1.54833	-1.42174

C	-0.18475	-0.83976	0.00000
H	0.48532	2.55626	1.48997
H	0.29817	1.02043	2.35038
H	-1.01782	1.62147	1.29444
H	0.48532	2.55626	-1.48997
H	-1.01782	1.62147	-1.29444
H	0.29817	1.02043	-2.35038
F	0.06569	-1.60915	1.08356
F	0.06569	-1.60915	-1.08356
F	-1.51951	-0.62328	0.00000

PF3_H_solvent
5

P	0.00007	-0.00010	0.30642
F	-0.34248	1.37054	-0.23283
F	-1.01599	-0.98165	-0.23292
F	1.35832	-0.38869	-0.23300
H	0.00037	-0.00033	1.69233

PF3_solvent
4

P	-0.00002	0.00007	0.52887
F	-0.87645	1.05747	-0.29377
F	-0.47771	-1.28773	-0.29390
F	1.35420	0.23015	-0.29378

PMe3_H_solvent
14

P	0.00008	0.00013	0.35140
C	1.37273	1.00959	-0.22089
C	0.18809	-1.69355	-0.22072
C	-1.56091	0.68373	-0.22074
H	1.13183	-2.09816	0.15186
H	0.19054	-1.69120	-1.31382
H	-0.64694	-2.29385	0.14741
H	2.30973	0.58870	0.15085
H	1.24910	2.03007	0.14846
H	1.37183	1.00721	-1.31398
H	-1.66470	1.70628	0.14904
H	-2.38310	0.06731	0.14967
H	-1.55889	0.68259	-1.31385
H	-0.00007	0.00039	1.74739

PMe3_solvent
13

P	0.00000	0.00000	0.59934
C	0.00000	1.62808	-0.27651
C	1.40996	-0.81404	-0.27651
C	-1.40996	-0.81404	-0.27651
H	0.88606	2.20120	0.01348
H	-0.88606	2.20120	0.01348
H	0.00000	1.49641	-1.36460
H	2.34933	-0.33325	0.01348
H	1.29593	-0.74820	-1.36460
H	1.46327	-1.86795	0.01348
H	-2.34933	-0.33325	0.01348
H	-1.46327	-1.86795	0.01348
H	-1.29593	-0.74820	-1.36460
